# Preoperative Manual Detorsion in Children With Intravaginal Testicular Torsion: A Single‐Center Experience

**DOI:** 10.1002/wjs.70455

**Published:** 2026-06-10

**Authors:** Maria Ruffoli, Anna Lavinia Bulotta, Giovanni Boroni, Susanna Milianti, Filippo Parolini, Luca Giacomo Tonegatti, Pierluigi Pedersini, Stefano Calza, Daniele Alberti

**Affiliations:** ^1^ Department of Pediatric Surgery Spedali Civili Children's Hospital Brescia Italy; ^2^ Department of Molecular and Translational Medicine Unit of Biostatistics and Bioinformatics University of Brescia Brescia Italy

**Keywords:** infant, ischemia, orchiopexy, spermatic cord torsion

## Abstract

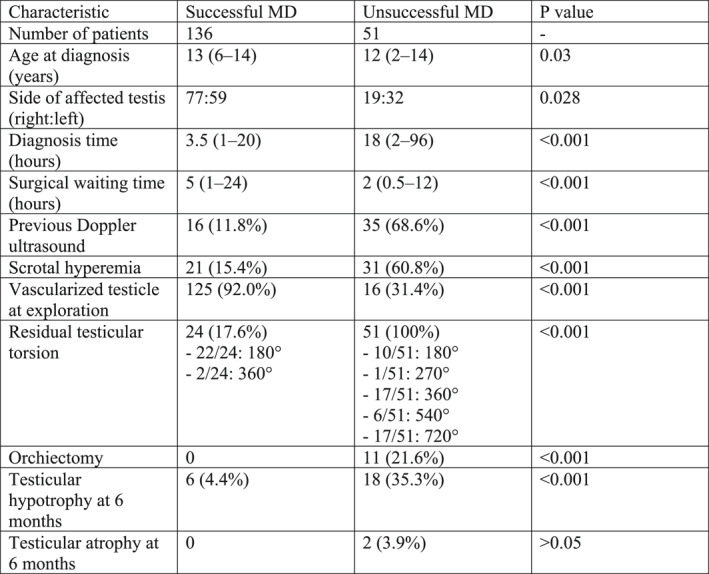

## 1 Introduction

Testicular torsion (TT) is a time‐critical pediatric emergency; testicular injury correlates with ischemia duration and degree of twist [1, 2]. Although scrotal exploration with bilateral orchiopexy is mandatory, preoperative manual detorsion (MD) can restore perfusion before surgery and may improve salvage [3]. We report outcomes of a standardized “MD‐first” protocol in children with intravaginal TT, with particular attention to age, time from symptom onset, residual torsion, and timing of definitive surgery.

## 2 Methods

We retrospectively reviewed consecutive patients < 14 years treated surgically for intravaginal TT at a single pediatric surgery unit (January 2015‐April 2023). Neonatal torsion, extravaginal torsion, and torsion of an undescended testis were excluded. TT was diagnosed clinically; color Doppler ultrasound was used in doubtful cases, particularly in children < 8 years, and selectively after MD. MD was attempted in the emergency department without anesthesia (initial medial‐to‐lateral “open‐book” rotation; reverse direction if pain increased). MD was considered successful when pain resolved rapidly with normalization of clinical findings; incomplete/partial relief prompted immediate exploration. All patients underwent scrotal exploration with contralateral fixation. Outcomes included age subgroup, symptom‐to‐diagnosis time, surgical waiting time, residual torsion at exploration, intraoperative viability, orchiectomy, and testicular hypotrophy/atrophy at 6 months.

## 3 Results

We included 187 patients (median age 13 years), and overall patient characteristics and outcomes are summarized in Table 1. MD was successful in 136/187 (72.7%). The youngest subgroup was small: all 8 children < 6 years belonged to the failure group. Symptom‐to‐diagnosis time was shorter in the successful group (median 3.5 vs. 18 h; *p* < 0.001). Manual detorsion success according to diagnosis time and age subgroup is detailed in Table 2. When diagnosis time exceeded 20 h, MD success was uncommon (2/27; 7.4%); all orchiectomies occurred after symptom duration > 12 h. At exploration, a vascularized testis was found in 125/136 (91.9%) after successful MD versus 16/51 (31.4%) after failed MD (*p* < 0.001). Residual torsion was detected in 24/136 (17.6%) in the successful group, but only two had persistent complete 360° torsion; both had only partial pain relief and underwent immediate surgery within 90 min. Orchiectomy was required in 11/187 (5.9%), all after failed MD (21.5% of failures; *p* < 0.001). At 6‐month follow‐up, 24/187 (12.8%) had testicular hypotrophy, predominantly after failed MD (4.4% vs. 35.3%; *p* < 0.001); the only two cases of complete atrophy occurred after failed MD.

**TABLE 1:** Patient characteristics and outcomes. Values shown as *n* or median (range).

Characteristic	Successful MD	Unsuccessful MD	*p* value
Number of patients	136	51	—
Age at diagnosis (years)	13 (6–14)	12 (2–14)	0.03
Side of affected testis (right:left)	77:59	19:32	0.028
Diagnosis time (hours)	3.5 (1–20)	18 (2–96)	< 0.001
Surgical waiting time (hours)	5 (1–24)	2 (0.5–12)	< 0.001
Previous Doppler ultrasound	16 (11.8%)	35 (68.6%)	< 0.001
Scrotal hyperemia	21 (15.4%)	31 (60.8%)	< 0.001
Vascularized testicle at exploration	125 (92.0%)	16 (31.4%)	< 0.001
Residual testicular torsion	24 (17.6%) − 22/24: 180° − 2/24: 360°	51 (100%) − 10/51: 180° − 1/51: 270° − 17/51: 360° − 6/51: 540° − 17/51: 720°	< 0.001
Orchiectomy	0	11 (21.6%)	< 0.001
Testicular hypotrophy at 6 months	6 (4.4%)	18 (35.3%)	< 0.001
Testicular atrophy at 6 months	0	2 (3.9%)	> 0.05

**TABLE 2:** Manual detorsion success according to diagnosis time and age.

	Successful MD	Unsuccessful MD
Diagnosis time < 20 h	116 (81.7%)	26 (18.3%)
Diagnosis time ≥ 20 h	2 (7.4%)	25 (92.6%)
Age < 6 years	0 (0%)	8 (100%)
Age ≥ 6 years	136 (76.0%)	43 (24.0%)

## 4 Discussion

In this cohort with systematic MD at diagnosis, MD succeeded in nearly three‐quarters of children and was associated with markedly higher intraoperative viability and lower orchiectomy rates. The very small number of younger children limits conclusions in that subgroup; however, all included cases were intravaginal torsions, so differences between intravaginal and extravaginal torsion cannot explain the age‐related findings in this series. Lower success in the youngest children may instead reflect delayed diagnosis, atypical presentation, and/or poorer compliance with a painful bedside maneuver, but these factors cannot be disentangled reliably in this retrospective dataset. Time from symptom onset was strongly associated with success: salvage is known to be highest within the first 12 h and to decline sharply thereafter, with very poor outcomes by 24 h; our data are consistent with this pattern, since all orchiectomies occurred after > 12 h and MD rarely succeeded after 20 h. MD did not replace surgery: residual torsion remained possible even with symptom improvement, supporting mandatory exploration and fixation [4, 5]. However, in children with complete pain relief and clinical normalization, early detorsion in the emergency department allowed conversion from immediate emergency exploration to semi‐emergent surgery, performed within 24 h of presentation in our protocol, whereas incomplete relief mandated urgent exploration.

## Author Contributions

**Anna Lavinia Bulotta:** conceptualization, writing – review and editing. **Giovanni Boroni:** supervision. **Maria Ruffoli:** conceptualization, investigation, writing – original draft. **Susanna Milianti:** supervision. **Daniele Alberti:** supervision. **Luca Giacomo Tonegatti:** supervision. **Pierluigi Pedersini:** supervision. **Filippo Parolini:** writing – review and editing. **Stefano Calza:** formal analysis.

## Funding

The authors have nothing to report.

## Ethics Statement

Institutional Review Board approval was obtained (NP6326/2024).

## Conflicts of Interest

The authors declare no conflicts of interest.

## Data Availability

The data that support the findings of this study are available on request from the corresponding author. The data are not publicly available due to privacy or ethical restrictions.
